# Tumor Immune Microenvironment Characterization of Primary Lung Adenocarcinoma and Lymph Node Metastases

**DOI:** 10.1155/2021/5557649

**Published:** 2021-07-10

**Authors:** Yuan Zhou, Xinying Shi, Huan Chen, Beibei Mao, Xue Song, Lingling Gao, Jiao Zhang, Ying Yang, Henghui Zhang, Guo Wang, Wei Zhuang

**Affiliations:** ^1^Department of Thoracic Surgery, Xiangya Hospital of Central South University, 410008 Changsha, Hunan, China; ^2^Beijing Genecast Biotechnology Co., Beijing, China; ^3^Department of Clinical Pharmacology, Xiangya Hospital of Central South University, 410008 Changsha, Hunan, China

## Abstract

**Background:**

The essential roles of the tumor microenvironment (TME) have been recognized during the initiation and progression of primary lung adenocarcinoma (LUAD). The aim of the present study was to delineate the immune landscape in both primary cancer and matched lymph node metastasis from a cohort of locally advanced stage LUAD patients with distinct outcomes.

**Methods:**

Formalin-fixed, paraffin-embedded samples were collected from 36 locally advanced LUAD patients. Transcriptome data of the tumor immune microenvironment were resolved using an immune oncology panel RNA sequencing platform. Bioinformatics approaches were used to determine the differentially expressed genes (DEGs), dysregulated pathways, and immune cell fraction between patients with early recurrence (ER) and late recurrence (LR).

**Results:**

Here, we showed that in primary cancer tissues, 23 DEGs were obtained between patients with ER and LR. Functional analysis revealed that the LR in LUAD patients may be associated with enriched gene sets belonging to the antigen presentation and MHC protein complex, innate immune response, and IFN-*γ* signaling pathways. Next, the transcriptome data were adopted to quantify immune cell fractions, indicating that high infiltration of mast cells and neutrophils was correlated with ER. Interestingly, similar findings were observed in metastatic lymph nodes from patients suffering from ER or LR. By analyzing the shared immune features of primary cancers and lymphatic metastases, we unraveled the prognostic value and joint utility of two DEGs, CORO1A and S100A8.

**Conclusions:**

In LUAD, the enrichment in antigen presentation, MHC protein complex, and IFN-*γ* signaling, and low infiltration of neutrophils in primary or metastatic nodules may be indications for a favorable prognosis. Integrated with bioinformatics approaches, transcriptome data of immune-related genes from formalin-fixed, paraffin-embedded (FFPE) samples can effectively profile the landscape of the tumor immune microenvironment and help predict clinical outcomes.

## 1. Introduction

Lung cancer is the most common malignancy worldwide and remains one of the leading causes of cancer-related deaths [1–4]. Lung cancer can be classified histologically as either small-cell lung cancer (SCLC) or non-small-cell lung cancer (NSCLC). In NSCLC, lung squamous cell carcinoma (LUSC) and lung adenocarcinoma (LUAD) are the two major subtypes that account for 80% of all lung cancers [1, 5]. Surgery is the primary treatment for early-stage lung cancer, and the 5-year survival rate after surgical resection was approximately 50% for early-stage LUAD and significantly decreased for locally advanced LUAD [1, 5, 6]. Usually, lymph nodes are among the first metastatic foci of lung carcinomas, and invasion into lymph vessels always implies an unfavorable prognosis and higher rates of locoregional recurrence and/or distant relapses [1, 7, 8]. However, locally advanced LUAD is a very heterogeneous disease, and some patients may suffer from rapid relapse after initial treatment, while others may show favorable progression-free survival (PFS) [9]. Therefore, clarifying the mechanisms of lymph node dissemination from LUAD would be of great importance for patient classification and prognosis evaluation.

When cancer cells start to form metastatic nodules, they need to communicate with the tumor microenvironment (TME), which is composed of various types of tumor-infiltrating lymphocytes (TILs), including T lymphocytes, B lymphocytes, neutrophils, macrophages, natural killer cells (NK cells), fibroblasts, and dendritic cells [7, 10]. During the metastatic process, cancer cells can trigger both an antitumor immune response (e.g., CD8+ cytotoxic T cells and NK cells) and immunosuppressive mechanisms, including regulatory T cells (Tregs), myeloid cells, and immunosuppressive stroma [11, 12]. Hence, molecular profiling of the primary tumors may help identify aberrations in both tumor cells and the instructed immune system in the TME.

Lymphatic metastasis is an early step in the metastatic cascade of cancer progression in LUAD, and its presence often reflects poor prognosis. As immune organs, lymph nodes may show conflicting roles in the cancer context. Initially, tumor antigens that drain to the lymph nodes can trigger an anticancer immune response and restrict metastatic nodule formation. However, as cancer progresses, immunomodulatory factors draining from the primary cancer may induce an immunosuppressive microenvironment in the lymph node that may eventually support metastatic outgrowth [13–15]. Together, these findings suggest that profiling of the lymph node microenvironment is also necessary to understand the mechanism of metastatic progression.

In recent years, a number of studies have explored transcriptome profiling in matched primary tumors and their metastatic nodules to characterize the primary tumors producing nodal metastases [16–18]. A recurrent observation was that the molecular features of nodal metastases resemble those of their matched primary tumors [19]. Based on the above evidence, we may infer that primary tumor and lymph node metastasis may share the malignant molecular features of cancer cells and some immunological factors in the induction process of immune tolerance.

In this study, we aimed to delineate the immune microenvironment in both primary cancer and matched lymph node metastasis from a cohort of locally advanced LUAD patients with distinct outcomes. Briefly, an immune oncology (IO) panel RNA sequencing platform was performed on formalin-fixed, paraffin-embedded (FFPE) specimens. Importantly, comprehensive characterization of the primary tumor and lymphatic metastases may help clarify the mechanisms by which cancer cells evade immune surveillance and identify TME factors with a potential prognostic value.

## 2. Material and Methods

### 2.1. Patient Enrollment and Study Design

Eligible patients were selected according to the following criteria: (1) surgery as the initial treatment and did not receive any preoperative therapy and refused postoperative adjuvant therapy; (2) postoperative pathology confirmed locally advanced LUAD with a pathological stage of T1-4N1-2M0; (3) patients from December 2014 to December 2016; (4) no other malignant tumors; (5) reach recurrence endpoint event: local lymph node recurrence/distant metastasis. The clinical data of the patients were reviewed, and all cases were stratified and analyzed according to the status of recurrence. Early recurrence (ER) was designated as patients with recurrence within 18 months after resection, and late recurrence (LR) was defined as recurrence occurring more than 18 months after resection.

The FFPE samples were retrieved from the tissue archive of Xiangya Hospital (Changsha, China). As summarized in Figure S1, primary tumor samples were collected from 36 locally advanced LUAD patients with recurrence. The institutional board of Xiangya Hospital gave explicit approval to the study, and all samples were obtained upon informed consent under an institutional protocol for tissue collection. Sections from the FFPE blocks were cut and stained with hematoxylin and eosin. Tumor tissue was confirmed by a qualified pathologist. Additional serial sections were cut for RNA extraction.

### 2.2. RNA Immune Oncology (IO) Profiling

The RNA IO profiling platform (Genecast Biotechnology, Beijing, China) is a unique 395-plex gene expression panel that quantifies 395 IO-related genes in human solid cancers in the following categories: tumor markers and essential signaling pathways, tumor-specific antigens, immunological response, infiltrating immune cells, and housekeeping (HK) genes. Briefly, RNA was extracted from FFPE tissues using the truXTRAC™ FFPE RNA Kit (Covaris, Inc., Woburn, MA). After purification, the RNA yield was quantified using a Qubit™ RNA HS Assay Kit (Thermo Fisher Scientific, Waltham, MA). Ten nanograms of RNA was reverse transcribed into cDNA, and targets were amplified with the primer pool targeting 395 genes. Barcode adaptors were ligated to partially digested amplicons. Purified libraries were quantified via an Agilent™ 2100 Bioanalyzer (Agilent, Santa Clara, CA) and then pooled in equimolar amounts prior to enrichment and template preparation using the Ion Chef™ system (Thermo Fisher Scientific, Waltham, MA). For each sample, 200 bp sequencing was performed on the Ion S5 530 chip (Thermo Fisher Scientific, Waltham, MA) to obtain 1-2 M reads. The absolute digital gene expression counts of all samples in the same run were automatically generated in the in-house bioinformatics pipeline. Only sequencing data meeting the quality control (QC) criteria for mapped reads, on-target reads, and mean reads were included in the study.

Gene expression normalization was performed as described previously [20]. A baseline expression profile for 10 HK genes was established based on average reads per million (RPM) counts. Each HK gene background-subtracted read was compared against the RPM profile from that internal control sample, which then gave rise to a fold change ratio for each HK gene: ratio of HK = absolute read count of HK/RPM profile of HK. Then, the normalization ratio for the particular sample was calculated using the median value of all HK ratios. The normalization ratio equals the median (all HK ratios). Next, the nRPM of all genes (G) of the specific sample (S) (nRPM(S,G)) was calculated as follows:

(1) $$\begin{array}{c} \text{nRPM}\left(\text{S},\text{G}\right)=\frac{\text{Background}-\text{subtracted read count}\left(\text{S},\text{G}\right)}{\text{Normalization ratio}\left(\text{S},\text{G}\right)}. \end{array}$$

### 2.3. Heat Map-Based Hierarchal Clustering and RNA Profile Analysis

Patients were divided into two groups (late recurrence group and early recurrence group) according to the median PFS time. The “limma” package was used to analyze the differentially expressed genes (DEGs) between patients with different groups. A heat map of DEGs was constructed using the log_2_ (nRPM +1) values. Each row represents a gene, and each column represents each patient. The leftmost patch indicates that 395 genes were annotated as 36 classes according to the signal/function pathway. Using R package “gene set variation analysis (GSVA)”, DEGs were analyzed and clustered according to 36 signal/function pathways between patients with late recurrence andearly recurrence. To analyze the correlation of gene set enrichment score and clinical outcomes, the corresponding Kaplan-Meier curves for PFS were then plotted, and the *p* value of the corresponding log-rank test was reported using the median of gene set enrichment score as the cutoff value. The RNA expression profiles of immune-related genes from primary or metastatic tumor samples were analyzed using gene set enrichment analysis (GSEA) and the Molecular Signatures Database (MSigDB) C5 gene sets.

## 3. Results

### 3.1. Clinical Characteristic

36 patients with primary tumor and lymph node metastasis performed RNA IO sequencing. There were 22 males and 14 females, with a median age of all patients was 58 years (range 36~77). Nine were N1 stage and 27 were N2 stage. The distributions of T stage were 33.3%, 50%, 8.3%, and 8.3% for T1, T2, T3, and T4, respectively. The percentages of differentiation level for poorly, moderate, and well were 30.6%, 55.6%, and 13.9%. The specified clinical data of the ER and LR groups are listed in Table 1.

### 3.2. Immune Oncology RNA Profiling of Primary LUAD with Tumor Recurrence

**Figure 1:**
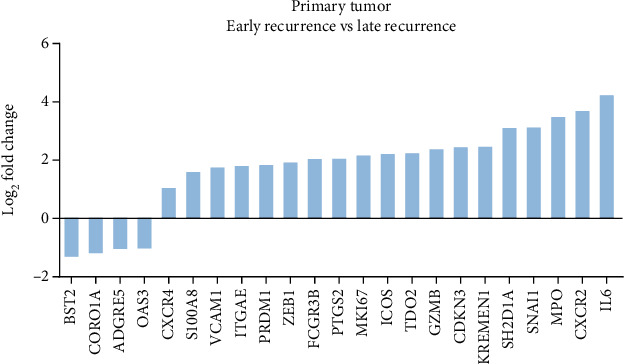
Immune oncology RNA profiling of primary lung adenocarcinoma. Waterfall plot of 23 differentially expressed genes (DEGs) between ER and LR subgroups in primary tumor. The genes were defined significantly expressed if the absolute fold change was ≥2 and the *p* value was <0.05.

To identify key TME players involved in lung cancer metastasis that may correlate with ER in locally advanced LUAD patients, we first explored the immune feature in the primary lung cancer tissue of 24 patients (Figure S1). The baseline characteristics of the included patients (e.g., gender, age median, T stage, N stage, and degree of differentiation) are presented in Supplementary Table S1. By comparing the transcriptome profiling data between 12 patients with ER and 12 with LR, we identified 23 DEGs (filtrating criteria: ∣fold change | ≥2, *p* < 0.05), which is illustrated in Figure 1. In the ER subgroup, 19 genes, including immunological factors [S100 calcium-binding protein A8 (S100A8), myeloperoxidase (MPO), interleukin 6 (IL6), granzyme B (GZMB), C-X-C motif chemokine receptor 2 (CXCR2), C-X-C motif chemokine receptor 4 (CXCR4), and so on], and tumor markers vascular cell adhesion molecule 1 (VCAM1), zinc finger E-box binding homeobox 1 (ZEB1), and snail family transcriptional repressor 1 (SNAIL) were upregulated, and 4 genes [Coronin 1A (CORO1A), 2′-5′-oligoadenylate synthetase 3 (OAS3), bone marrow stromal cell antigen 2 (BST2), and adhesion g protein-coupled receptor E5 (ADGRE5)] were downregulated versus that of the LR subgroup.

### 3.3. Functional Characterization of the Immune Microenvironment of Primary LUAD Samples

**Figure 2:**
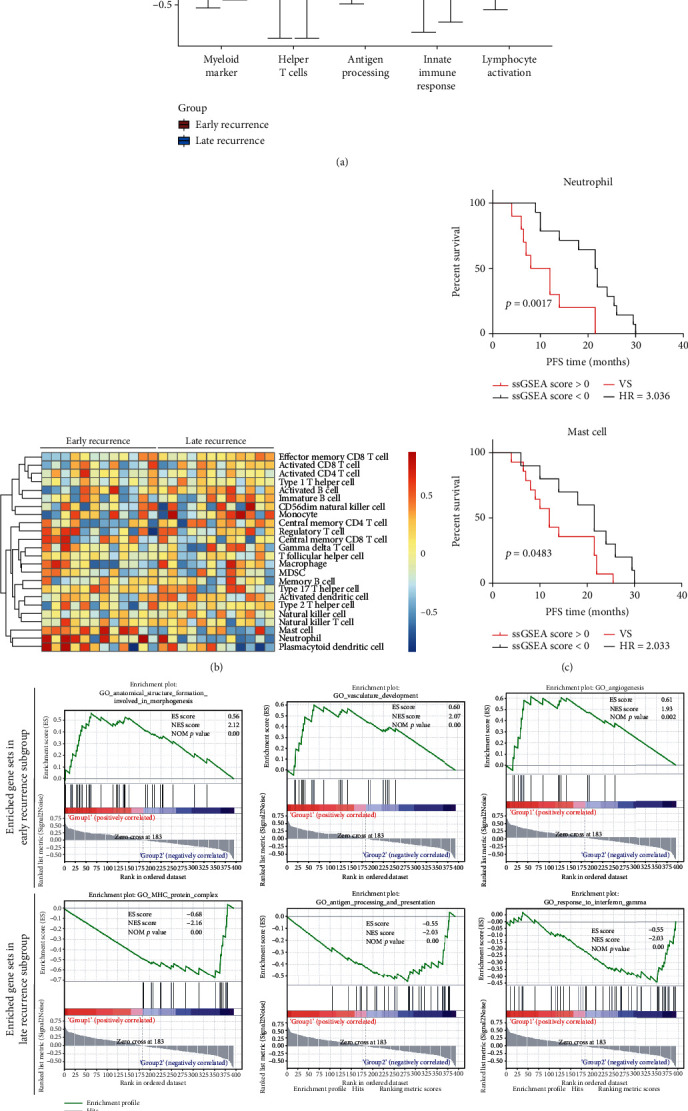
Differentially expressed immunological pathways and infiltrating immune cell types of primary LUAD. (a) Boxplot of GSVA enrichment score of immune-related pathways in primary LUAD samples between ER and LR subgroups. (b) Heat map of ssGSEA enrichment score of immune cell fraction in primary LUAD samples between ER and LR subgroups. (c) Progression-free survival (PFS) analysis of LUAD patients stratified by infiltration levels of neutrophils and mast cells. (d) GSEA of RNA IO dataset from primary lung adenocarcinoma with tumor recurrence.

Further functional study by using GSVA analysis revealed that antigen processing, innate immune response, and lymphocyte activation pathways were upregulated in LUAD patients with better prognosis (LR), while pathways regarding myeloid cells and helper T cells were elevated in patients with ER (Figure 2(a)). Moreover, the GSVA immune cell population revealed that neutrophils (*p* = 0.002) and mast cells (*p* = 0.008) were enriched in the ER group (Figure 2(b)), and higher neutrophil or mast cell infiltration was correlated with shorter PFS (neutrophil, *p* = 0.0017; mast cell, *p* = 0.0483) (Figure 2(c)). In addition, we also performed GSEA analysis on the RNA IO profiling data, which is a widely adopted overrepresentation assay to determine if given sets of genes are differentially expressed in a specific phenotype [21]. As expected, enriched gene sets in the ER subgroup were anatomic structure formation involved in morphogenesis, vasculature development, and angiogenesis (Figure 2(d), upper panel). On the other hand, gene sets enriched in the LR subgroups were MHC protein complex, antigen processing and presentation, and interferon gamma (IFN-*γ*) (Figure 2(d), lower panel). Overall, the locally advanced LUAD patients showed distinct immunological microenvironments that may reflect different tumor biology leading to ER or LR.

### 3.4. Immune Oncology RNA Profiling of Metastatic Lymph Nodes in LUAD Patients

**Figure 3:**
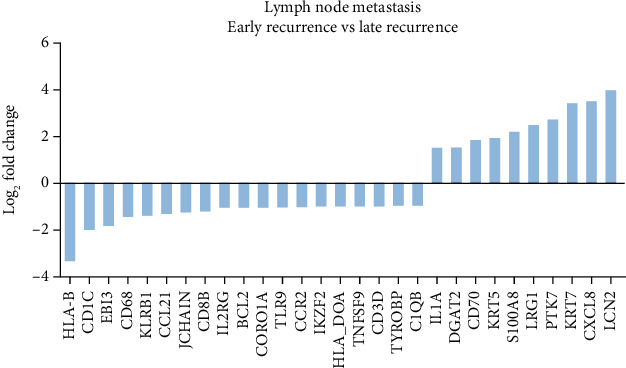
Immune oncology RNA profiling of lymph node metastases. Waterfall plot of 29 differentially expressed genes (DEGs) between ER and LR subgroups in metastatic lymph node samples. The genes were defined significantly expressed if the absolute fold change was ≥2 and the *p* value was <0.05.

It is becoming increasingly apparent that multiple changes also occur in lymph nodes during the process of metastasis formation [13], and their relevance to prognosis in LUAD patients is worthy of further investigation. First, the correlation between clinical characteristics and LUAD recurrence was analyzed, and no significant correlation was found between the clinical outcomes and major clinical parameters, including gender, age, tumor T stage, or degree of differentiation (Supplementary Table S2), suggesting that the heterogeneity of the patients with distinct clinical outcomes may result from different molecular features. Next, the DEGs between lymph node metastases from the ER and LR subgroups were obtained using the RNA IO panel sequencing platform. There were 19 overexpressed genes and 10 downregulated genes in the LR subgroup when compared with the ER subgroup, as depicted in the waterfall plot (Figure 3). Specifically, the upregulated genes in the metastatic lymph node of LUAD patients with favorable prognoses include CORO1A, interleukin 2 receptor subunit gamma (IL2RG), CD8b molecule (CD8B), TYRO protein tyrosine kinase binding protein (TYROBP), and major histocompatibility complex, class I, B (HLA-B), while S100A8, interleukin 1 alpha (IL1A), protein tyrosine kinase 7 (PTK7), keratin 7 (KRT7), and keratin 5 (KRT5) were overexpressed in the ER subgroup.

### 3.5. Functional Characterization of the Immune Microenvironment of Primary LUAD Samples

To determine the functional difference between lymph node metastases in the ER subgroup and those belonging to the LR subgroup, we also performed GSVA and GSEA functional analysis (Figure 3). First, the GSVA study revealed that the T cell regulation pathway was enriched in the ER subgroup, while the NK cell marker pathway was enriched in the LR subgroup (Figure 3(a)). Second, the GSEA functional study showed that late LUAD recurrence may be correlated with more pathways, including negative regulation of growth, the T cell receptor signaling pathway, antigen binding, positive regulation of IFN-*γ* production, lymphocyte costimulation, and the MHC protein complex (Figure 3(c)). Third, the GSVA immune cell population analysis demonstrated that higher infiltration of activated CD8+ T cells, effector memory CD8+ T cells, and macrophages and lower infiltration of neutrophils were identified in the LR subgroup compared with the ER subgroup (Figure 3(b)). These observations indicate that abundant neutrophils, loss of MHC binding and antigen presentation, and decreased cytotoxic activity by T cells or NK cells in the lymphatic metastases may collectively contribute to the unfavorable prognosis of LUAD patients.

### 3.6. Shared Immune Features in Matched Primary Cancer and Metastatic Lymph Nodes

**Figure 4:**
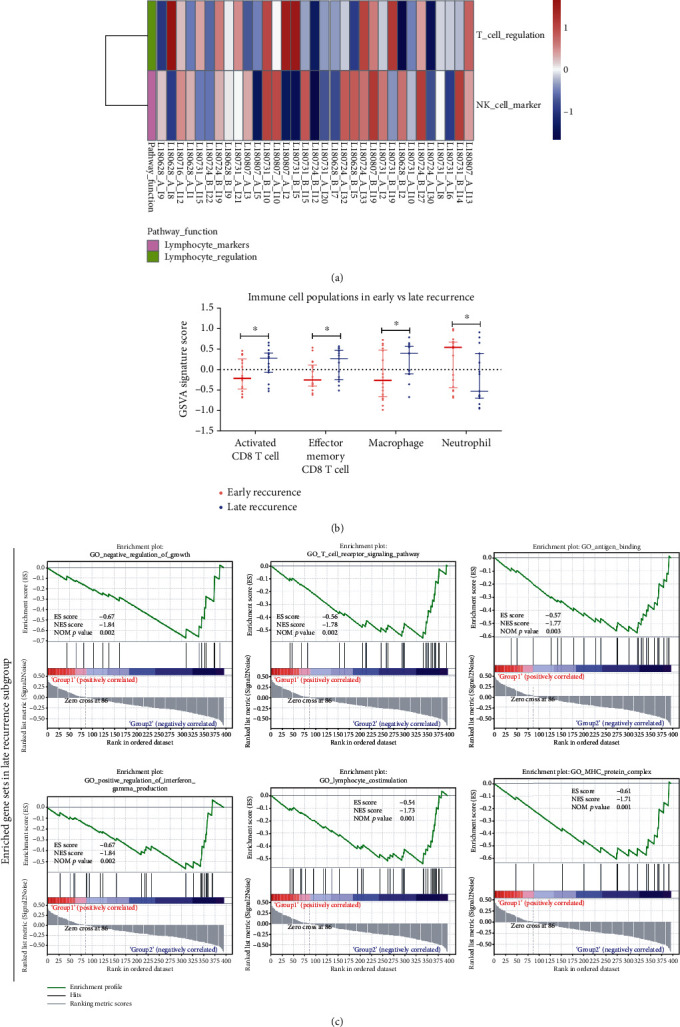
Differentially expressed immunological pathways and infiltrating immune cell types of lymph node metastases. (a) Heat map of GSVA enrichment score of immune-related pathways in metastatic lymph node samples between ER and LR subgroups. (b) Scatter plot of the ssGSEA enrichment score of the immune cell fraction in metastatic lymph node samples between ER and LR subgroups. (c) GSEA of RNA IO dataset from lymph node metastases.

**Figure 5:**
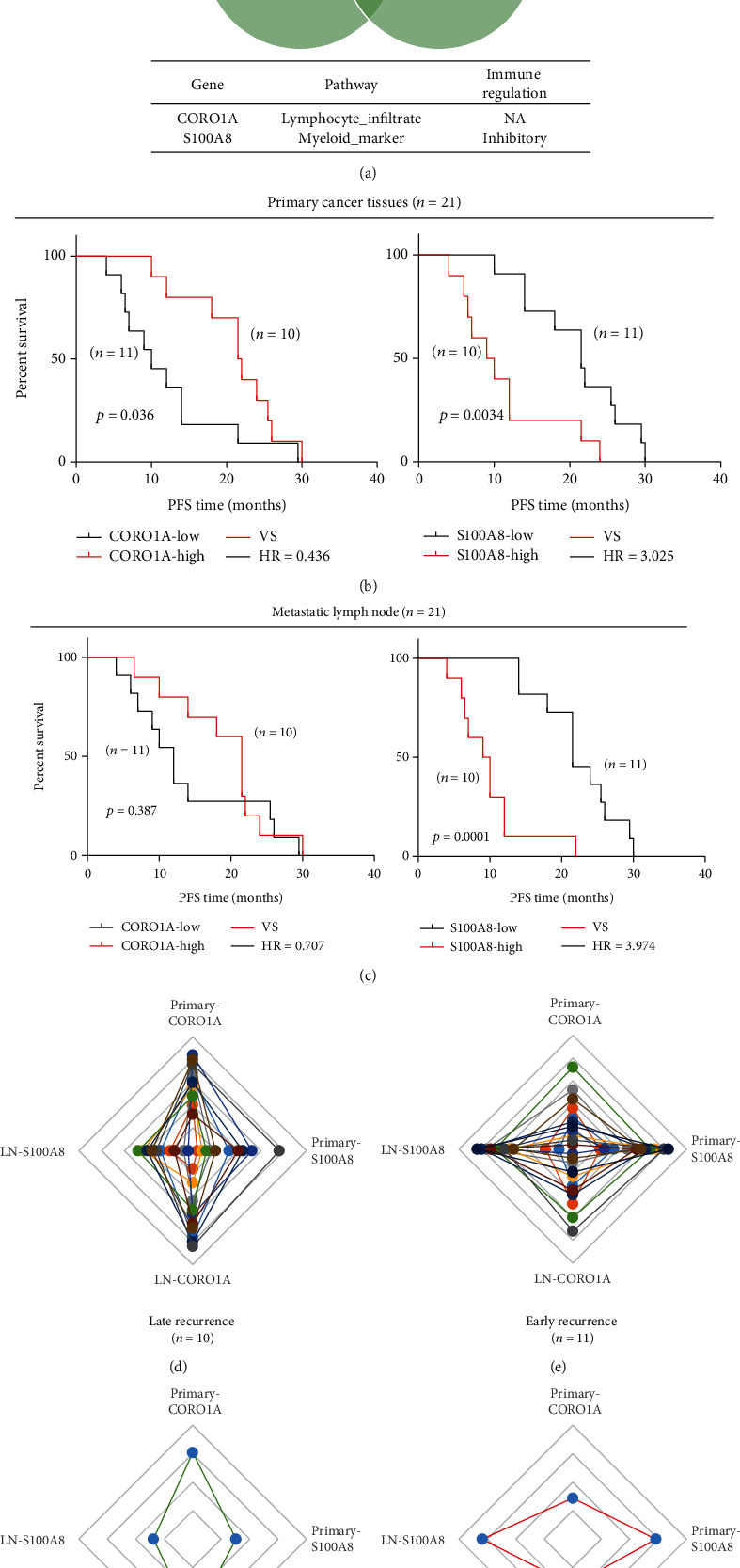
Expressions of CORO1A and S100A8 in primary and metastatic lymph nodes characterize tumor recurrence. (a) Shared DEGs in primary cancer and metastatic lymph node samples. (b, c) Patients (*n* = 21) who had transcriptome data from matched primary cancer and metastatic lymph node samples were subjected to Kaplan–Meier survival analysis. PFS survival of these patients was stratified by expression levels of CORO1A (b) and S100A8 (c). (d, e) Expression levels of CORO1A and S100A8 in primary cancer and metastatic lymph nodes in each patient within the ER and LR subgroups. (f, g) Average expression levels of CORO1A and S100A8 in primary cancer and metastatic lymph nodes in ER and LR patients.

As revealed in Figures 1–4, we observed multiple shared immune features enriched in matched primary cancer and metastatic lymph node in the patients with LR. Specifically, the MHC protein complex, IFN-*γ* signaling, antigen binding, and presentation pathways were enriched in the LR subgroup when compared with that of the ER subgroup (Figures 2 and 4). Moreover, infiltration of neutrophils in either primary cancer or metastatic lymph node may be correlated with ER in LUAD patients (Figures 2 and 4). Intriguingly, we found two shared DEGs (1 upregulated and 1 downregulated) by cross comparing the gene list obtained from Figure 1 (DEGs in primary tissue) and Figure 3 (DEGs in metastatic lymph tissue), suggesting that the two DEGs may collectively contribute to the tumor biology and immune functions in patients with distinct clinical outcomes (Figure 5(a)).

Next, to demonstrate the prognostic value of the two genes in matched primary and metastatic lymph nodes in LUAD patients, we performed Kaplan-Meier analysis on the 21 patients (ER, *n* = 11; LR, *n* = 10). As Figures 5(b) and 5(c) show, increased expression levels of CORO1A and decreased levels of S100A8 in the primary tumor or lymphatic metastases were correlated with longer PFS. Furthermore, for each of the 21 patients who had complete RNA IO profiling data of matched primary and metastatic lymph nodes, individuals within the ER or LR subgroup showed different expression patterns according to the expression levels of the two genes (Figures 5(d)–5(g)).

**Figure 6:**
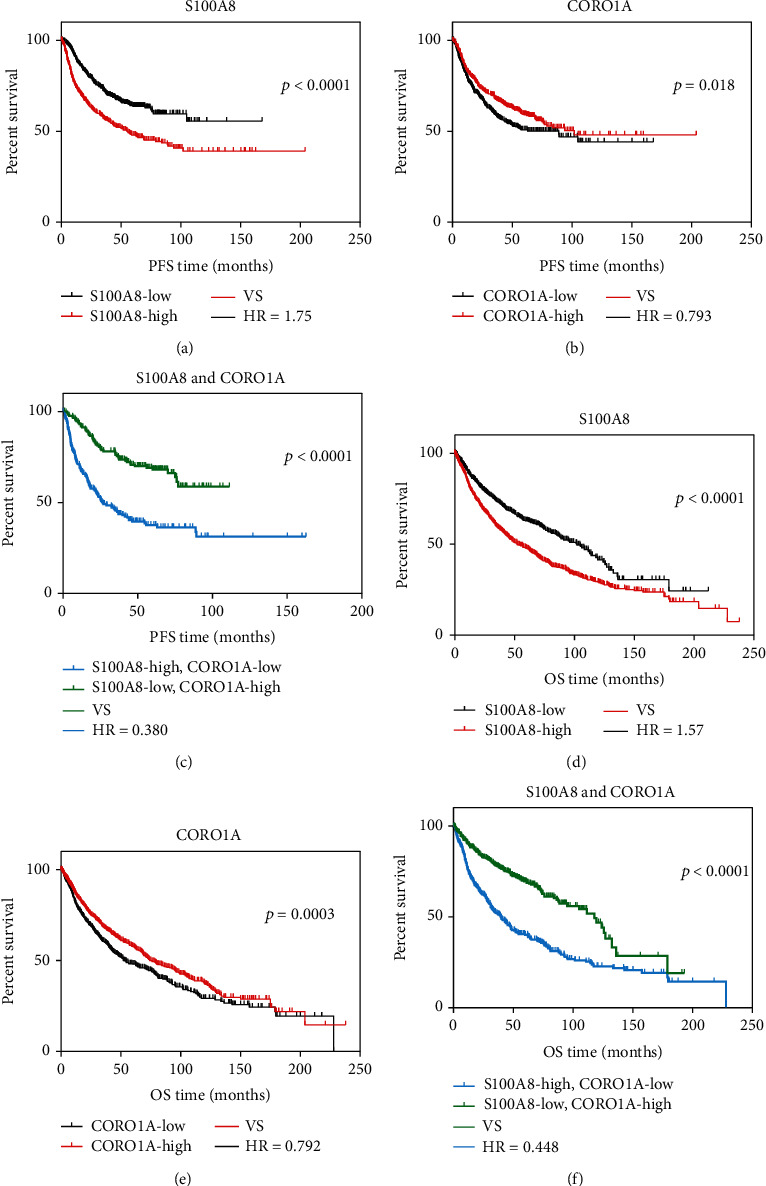
Determination of the prognostic and combinatorial values of CORO1A and S100A8 using the KMplotter dataset. (a–c) In the KMplotter online dataset, PFS curves were plotted according to the expression levels of S100A8 (a), CORO1A (b), and the joint value of both (c). (d–f) In the KMplotter online dataset, overall survival curves were plotted according to the expression levels of S100A8 (d), CORO1A (e), and the joint value of both (f). The high or low expression level of each gene was determined using the autoselect best value via the online tool.

Additionally, to validate the prognostic value of the two genes, we obtained gene expression data of primary lung cancer tissue from the KMplotter online tool http://kmplot.com/analysis/index.php?p=service&cancer=lung. Kaplan-Meier analysis revealed that high levels of the CORO1A gene and low levels of the S100A8 gene are correlated with longer PFS and OS (Figure 6). Intriguingly, when combining the two biomarkers, patients within the S100A8-low/CORO1A-high subgroup showed longer median PFS (mPFS, undefined) and median OS (mOS, 118 months) when compared with the S100A8-high/CORO1A-low subgroup (mPFS, 26.71 months; mOS, 39.6 months, *p* < 0.0001 for both comparisons). Collectively, the above data indicate that S100A8 and CORO1A are two immune-related genes with a potential prognostic value.

## 4. Discussion

In the past decade, the essential roles of the TME in the initiation and progression of primary LUAD have been recognized [22]. Immunological changes occurring during the process of cancer procession and metastatic nodule formation include the expansion of immunosuppressive cells, expression of cytokines and chemokines, lymphangiogenesis, and blood vessel remodeling [23, 24]. Indeed, much attention has been paid to TME characterization of the primary tumor in previous reports [25, 26]. However, the TME landscape in lymphatic metastases and its prognostic relevance in locally advanced LUAD patients may have been underestimated. Here, in the present investigation, we comprehensively characterize the immune features of the primary tumor and matched lymph node metastases in a cohort of locally advanced LUAD patients who experienced early or LR.

Evidence suggests that molecular characterization of the TME landscape has the potential to uncover new biomarkers and molecular features that impact cancer progression [22, 27, 28]. We first analyzed the immunological transcriptome data from primary LUAD patients with ER or LR. As shown in Figure 2, a better prognosis in the LUAD patients may be associated with enriched gene sets belonging to the antigen presentation and MHC protein complex, innate immune response, and IFN-*γ* signaling pathways. More interestingly, by comparing the RNA profiling data of the lymphatic metastases between the ER and LR subgroups, antigen binding and presentation, MHC protein complex, and IFN-*γ* signaling were also enriched in the LR subgroup (Figure 4). In accordance with our data, decreased expression of MHC antigen in lung cancers has been reported [29–31], and MHC class I antigen expression level is associated with prolonged survival of patients with lung cancer [32, 33]. Several pathways have been proposed to explain the impaired expression of MHC molecules and antigen presentation, including loss of transporter of antigen presentation (TAP) or loss of *β*2 microglobulin [34, 35]. Furthermore, the present study also presented evidence of the potential relevance of the IFN-*γ* signaling pathway in defining the malignancy of lung cancer (Figures 2 and 4). As an essential component within the TME, IFN-*γ* produced by T cells and NK cells has been shown to activate cytotoxic responses and inhibit tumor progression [36, 37]. Taken together, our data confirmed the previously proposed notion that immune evasion of cancer cells develops via a series of pathways, including defects in antigen presentation, impaired MHC expression, and defects in IFN-*γ* signaling [22].

Being recognized as a major player involved in the progression and metastatic process of cancer, infiltrating immune cells exert various biological functions, even among cancer types [10, 38–40]. Therefore, profiling of TILs in primary cancer and metastatic nodules can shed light on the underlying mechanisms and potential therapeutic strategies [41]. Recently, transcriptome data have been adopted to quantify immune cell fractions by using bioinformatics approaches based on a set of immune-specific marker genes or expression signatures [42]. Here, we assess the RNA IO profiling data and examine the composition of different immune cell types in both primary LUAD samples and metastatic lymph nodes. Increased infiltration of mast cells and neutrophils was observed in the primary cancer tissue of patients with ER (Figure 2(b)), and the higher abundance of the two cell types was associated with shorter PFS in this cohort (Figure 2(c)). Notably, higher levels of neutrophils and lower levels of CD8+ T cells and macrophages were found in lymphatic metastatic nodules in the ER subgroup when compared to that of the LR subgroup (Figure 4), suggesting defects in both innate and adaptive immunities in these locally advanced patients who suffer from early recurrence. Intriguingly, a recent study demonstrated that neutrophils are the most abundant immune cell type in NSCLC specimens and implicated neutrophils as potential immune suppressive factors in cancer progression [43]. In accordance with our finding, the neutrophil transcript signature has been shown to be a strong predictor of mortality in a large cohort of NSCLC patients [44]. The novel finding is that higher infiltration of neutrophils in the primary cancer and metastatic lymph node in patients with unfavorable prognosis may provide potential therapeutic strategies.

Here, we also analyzed the shared immune features in matched primary cancer and metastatic lymph nodes. Our data demonstrated the prognostic value and joint utility of two genes, CORO1A and S100A8 (Figures 5 and 6). CORO1A, which encodes a Trp-Asp (WD) repeat protein, has been implicated in diverse cellular processes. CORO1A deficiency may compromise homeostasis and T cell function caused by defective T cell receptor (TCR) signaling [45, 46]. On the other hand, S100A8 is a calcium-binding protein that is highly expressed in neutrophils and monocytes, and it could induce neutrophil adhesion to fibrinogen in vitro during inflammatory conditions [47]. In the context of cancer, S100A8 may facilitate the homing of tumor cells to the premetastatic niche [48]. Therefore, the two potential prognostic biomarkers jointly reflect the function of T cells and neutrophils in the TME of lung cancer. Additionally, the prognostic value and combinatorial value of the two genes were further validated in the KMplotter online database, suggesting further utility in the assessment of recurrence risk in lung cancer.

## 5. Conclusions

The comprehensive characterization of the TME landscape in matched primary lung cancer and lymph node metastases unveils the multifaceted role of the immune system during the metastatic process of lung cancer progression and offers potential prognostic biomarkers for patient stratification. Further studies are still needed to define the exact role of these immune features and the underlying mechanism.

## Figures and Tables

**Table 1:** Baseline characteristics of 36 LUAD patients.

Characteristic	Total (*N* = 36)	LR (*n* = 17)	ER (*n* = 19)
Gender			
Male	22 (61.1)	12 (33.3)	10 (27.8)
Female	14 (38.9)	5 (13.9)	9 (25)
Age			
≤58	18 (50)	9 (25)	9 (25)
>58	18 (50)	8 (22.2)	10 (27.8)
N stage			
N1	9 (25)	6 (16.7)	3 (8.3)
N2	27 (75)	11 (30.6)	16 (44.4)
T stage			
T1	12 (33.3)	7 (19.4)	5 (13.9)
T2	18 (50)	8 (22.2)	10 (27.8)
T3	3 (8.3)	1 (2.8)	2 (5.6)
T4	3 (8.3)	1 (2.8)	2 (5.6)
Differentiation			
Poorly	11 (30.6)	4 (11.1)	7 (19.4)
Moderate	20 (55.6)	10 (27.8)	10 (27.8)
Well	5 (13.9)	3 (8.3)	2 (5.6)

## Data Availability

The datasets used and/or analyzed during the current study are available from the corresponding authors on reasonable request.

## Abbreviations

TME: Tumor microenvironment
LUAD: Lung adenocarcinoma
DEGs: Differentially expressed genes
ER: Early recurrence
LR: Late recurrence
FFPE: Formalin-fixed, paraffin-embedded
SCLC: Small-cell lung cancer
NSCLC: Non-small-cell lung cancer
LUSC: Lung squamous cell carcinoma
PFS: Progression-free survival
TILs: Tumor-infiltrating lymphocytes
NK: Natural killer
IO: Immune oncology
HK: Housekeeping
QC: Quality control
RPM: Reads per million
GSVA: Gene set variation analysis
GSEA: Gene set enrichment analysis
MSigDB: Molecular Signatures Database.

## Supplementary Materials

Supplementary Materials Table S1: baseline characteristics of the LUAD patients (*n* = 24), from whom the primary cancer tissue was analyzed. Table S2: baseline characteristics of the LUAD patients (*n* = 33), from whom the lymph node metastases were analyzed.
