# Supplementary material for: Tumor Immune Microenvironment Characterization of Primary Lung Adenocarcinoma and Lymph Node Metastases
Source: Biomed Res Int. 2021 Jul 10;2021:5557649. doi: 10.1155/2021/5557649 (PMC8292094; doi:10.1155/2021/5557649)
Supplement: Supplementary Materials — Table S1: baseline characteristics of the LUAD patients (n = 24), from whom the primary cancer tissue was analyzed. Table S2: baseline characteristics of the LUAD patients (n = 33), from whom the lymph node metastases were analyzed. [file 5557649.f1.zip › FigureS1-Supplemental Files.pptx]

## Slide 1
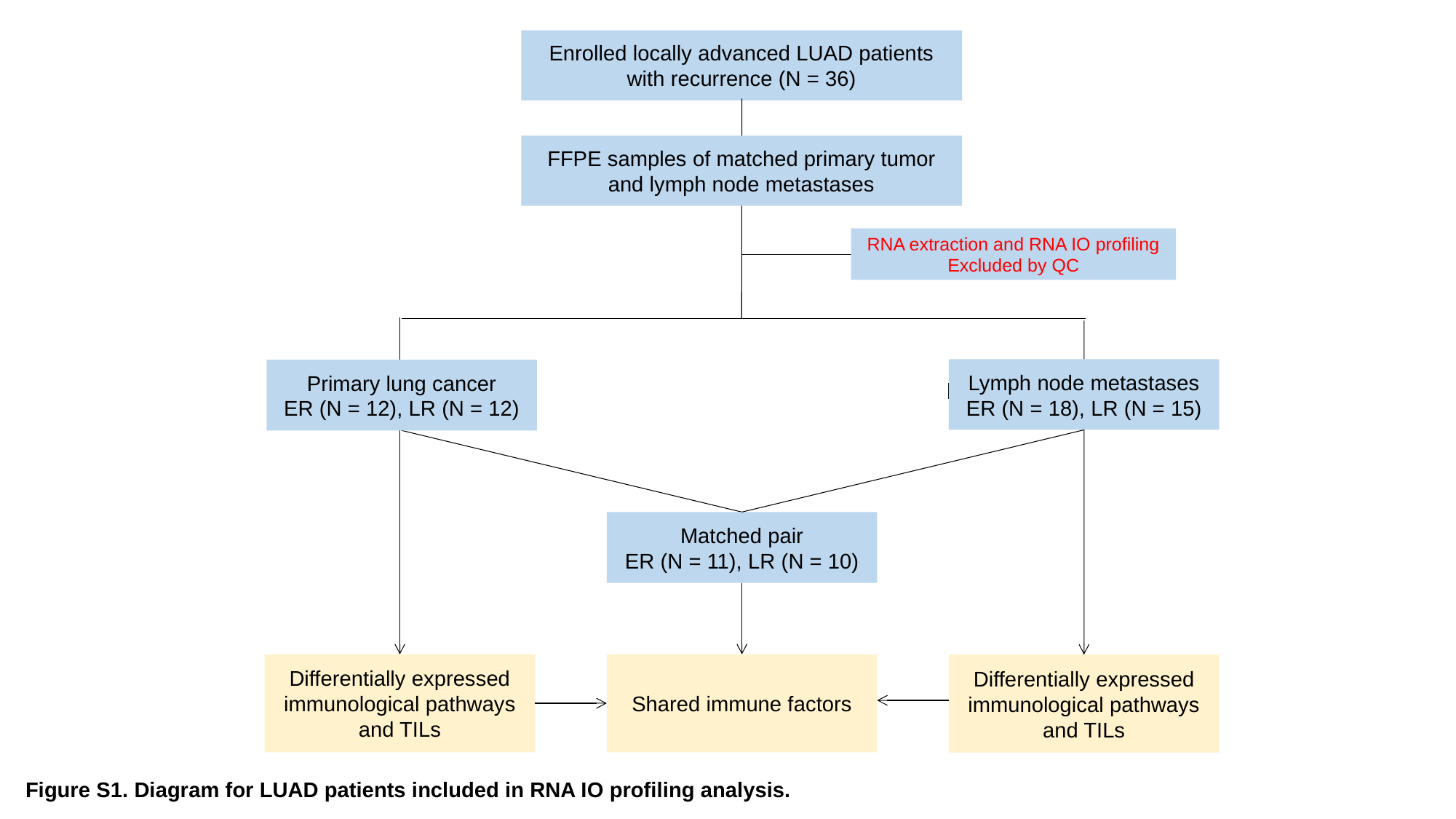

Enrolled locally advanced LUAD patients with recurrence (N = 36)
FFPE samples of matched primary tumor and lymph node metastases
RNA extraction and RNA IO profiling
Excluded by QC
Lymph node metastases
ER (N = 18), LR (N = 15)
Primary lung cancer
ER (N = 12), LR (N = 12)
Matched pair
ER (N = 11), LR (N = 10)
Differentially expressed immunological pathways and TILs
Shared immune factors
Differentially expressed immunological pathways and TILs
Figure S1. Diagram for LUAD patients included in RNA IO profiling analysis.
