# Supplementary material for: Tumor Immune Microenvironment Characterization of Primary Lung Adenocarcinoma and Lymph Node Metastases
Source: Biomed Res Int. 2021 Jul 10;2021:5557649. doi: 10.1155/2021/5557649 (PMC8292094; doi:10.1155/2021/5557649)
Supplement: Supplementary Materials — Table S1: baseline characteristics of the LUAD patients (n = 24), from whom the primary cancer tissue was analyzed. Table S2: baseline characteristics of the LUAD patients (n = 33), from whom the lymph node metastases were analyzed. [file 5557649.f1.zip › new Table S2-Supplemental Files.docx]

**Table S2.** Baseline characteristics of the LUAD patients (n=33), from whom the lymph node metastases were analyzed.

|  | **LR (n=15)** | **ER (n=18)** | **P value** |
| --- | --- | --- | --- |
| **Gender** |  |  | 0.172 |
| Male | 11 | 9 |  |
| Female | 4 | 9 |  |
| **Age** |  |  | 0.373 |
| ≤58 | 9 | 8 |  |
| >58 | 6 | 10 |  |
| **N stage** |  |  | 0.239 |
| N1 | 6 | 3 |  |
| N2 | 9 | 15 |  |
| **T stage** |  |  | 0.729 |
| T1 | 6 | 4 |  |
| T2 | 7 | 10 |  |
| T3 | 1 | 2 |  |
| T4 | 1 | 2 |  |
| **Differentiation** |  |  | 0.786 |
| Poorly | 3 | 6 |  |
| Moderate | 10 | 10 |  |
| Well | 2 | 2 |  |
